# Reproducibility of a novel computed-tomography based measurement of renal papillary density in the Framingham Heart Study

**DOI:** 10.1186/s13104-015-1784-6

**Published:** 2015-12-22

**Authors:** Aaron J. Yeoh, Joe Massaro, Caroline S. Fox, Udo Hoffmann, Brian H. Eisner, Gearoid M. McMahon

**Affiliations:** National Heart, Lung and Blood Institute’s Framingham Heart Study, 73 Mt. Wayte Avenue, Suite 2, Framingham, MA 01702 USA; Department of Biostatistics, Boston University School of Public Health, Boston, MA USA; Division of Endocrinology and Metabolism, Brigham and Women’s Hospital, Boston, MA USA; Department of Medicine, Boston University School of Medicine, Boston, MA USA; Department of Urology, Massachusetts General Hospital, Boston, MA USA; Renal Division, Brigham and Women’s Hospital, Boston, MA USA

**Keywords:** Chronic kidney disease, Computed tomography, Renal papillary density

## Abstract

**Background:**

Renal papillary calcification is a compelling candidate risk factor for chronic kidney disease (CKD) and nephrolithiasis. Renal papillary density (RPD), as assessed by computed tomography (CT), is a potential marker for calcification that has not been well studied. We developed a protocol to measure RPD using CT scans and assessed its reproducibility in participants from the Framingham Heart Study.

**Methods:**

We assessed RPD of right kidneys from a single abdominal CT slice in 100 representative participants from the Framingham Heart Study (47 % female, mean age 59.9 years) using a novel protocol. We selected the kidney slice with the most open sinus space and assessed RPD using the average of three 20 mm^2^ ellipses from upper, middle and lower papillary regions. Two different readers performed RPD measurements and the first reader repeated all measurements to determine both intra- and inter-reader reproducibility, respectively.

**Results:**

Of 100 total individuals included in the replication dataset, six were excluded for poor scan quality. Average RPD across all individuals was 48.7 ± 4.7 (range 38.7–61.7) Hounsfield Units (HU). The intra- and inter-reader correlation coefficients were 0.86 and 0.79, respectively. Bland–Altman analysis suggested no systematic bias between the different reads.

**Conclusion:**

Measuring RPD is practical and reproducible using MDCT scans from a small sample of a community-based cohort.

## Background

Chronic kidney disease (CKD) is an important public health problem that affects 10–15 % of adults in the United States [1, 2]. CKD is independently associated with cardiovascular disease (CVD) and an increased risk of mortality [3, 4]. Identification of the risk factors associated with CKD earlier in the progression of the disease could help elucidate the mechanism of CKD development and shed light on potential target interventions that could delay progression and reduce future complications.

Renal papillary calcification is a potential candidate risk factor for CKD. Nephrolithiasis is an independent risk factor for CKD and end-stage renal disease [5, 6]. Biopsies of renal papillary tissue in patients with nephrolithiasis show that even modest, subclinical calcification is associated with interstitial fibrosis, tubular obstruction and scarring [7, 8]. Renal papillary density (RPD) assessed with computed tomography (CT) is a potential marker of papillary calcification. Substantially higher RPD was observed in individuals with a history of kidney stones and RPD is a risk factor for future kidney stones [9–12]. Notably, biopsy-verified papillary calcification and increased RPD are observed in both unaffected and affected kidneys of individuals with nephrolithiasis [12]. These findings suggest that increased renal papillary calcification can arise in the absence of clinical kidney stones and is suggestive of systemic metabolic derangements. Thus, RPD may be an ideal, non-invasive surrogate marker for renal papillary calcification.

The feasibility and reproducibility of measuring RPD using CT has not been well studied. A robust technique for an accurate, non-invasive measurement of RPD could provide important information about current kidney health and provide greater information about the risk of developing CKD. We aimed to develop a novel reproducible protocol to measure RPD using CT scans in participants from the Framingham Heart Study cohort.

## Results

### Study sample characteristics

Of the 100 participants in the reproducibility sample, six were excluded due poor image quality or the lack of visible kidney sinus. Characteristics of the remaining 94 participants are presented in Table 1. The sample was 47 % women and the mean age was 59.9 ± 13.0 years. The mean BMI was 27.8 ± 4.6. The mean RPD measurement for the first reader was 48.7 ± 4.7 HU (range 38.7–61.7).

**Table 1:** Characteristics of participants at the baseline examination

Risk factor	Mean/Median/N	SD/IQR/%
Female	46	47 %
Age, years	59.9	13.0
Diabetes mellitus	10	10.2 %
Systolic blood pressure, mmHg	125.5	16.7
Diastolic blood pressure, mmHg	73.9	9.8
Hypertension Rx	32	32.7 %
Hypertension	38	38.8 %
BMI, kg/m^2^	27.8	4.6
HDL cholesterol, mg/ml	52.6	14.3
Triglycerides, mg/ml	124.9	76.0–163.0
Current smoker	12	12.2 %
eGFR, ml/min/1.73 m^2^	83.8	20.4
Dipstick proteinuria	13	13.5 %
Prevalent CKD	5	5.2
Prevalent CVD	9	9.2

### Intra- and inter-reader reproducibility

**Fig. 1:**
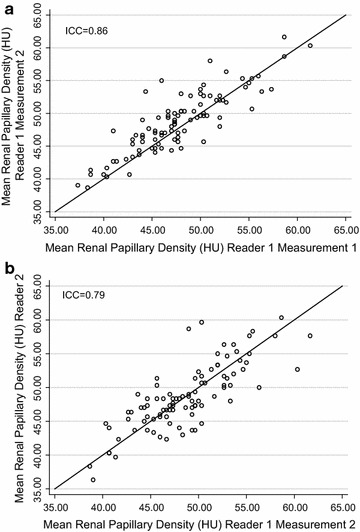
Plot comparing the mean RPD for the intra-reader replication (**a**) and inter-reader replication (**b**). Linear regression of mean RPD for intra-reader and inter-reader measurements. Intra-class correlation coefficients were 0.86 and 0.79 respectively

Intra- and inter-reader RPD measurement comparisons are plotted in Fig. 1a, b, respectively. For our single slice protocol, the intra-reader intra-class correlation coefficient was 0.86 while the inter-class correlation was 0.79.

**Fig. 2:**
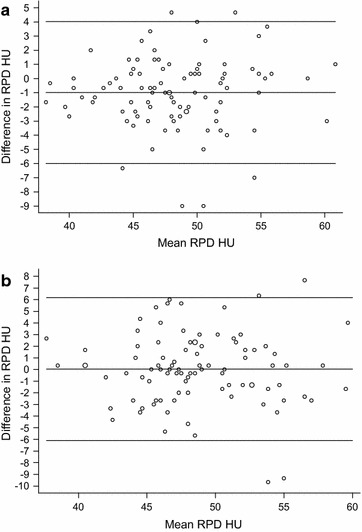
Bland–Altman plot showing the intra-reader and inter-reader repeated RPD measurements. The mean difference between the two intra-reader reads was 0.98 (95 % CI 0.47–1.50). Pitman’s test of difference in variance was negative suggesting little bias (p = 0.66) (**a**). The mean difference between the two inter-reader reads was 0.04 (95 % CI −0.59 to 0.67). Pitman’s test of difference in variance was negative (p = 0.64) (**b**)

The Bland–Altman comparison of the intra-reader repeated measurements is presented in Fig. 2a. The overall mean difference was 0.98 with lower and upper 95 % confidence intervals of 0.47 and 1.50, respectively. Pitman’s Test of Difference in Variance showed no systematic bias between intra-reader repeated measurements (p = 0.66). The Bland–Altman plot of the inter-reader measurements is presented in Fig. 2b. The overall mean difference between the two readers was 0.043 (95 % CI −0.59 to 0.67). Pitman’s Test of Difference in variance showed no systematic bias between two different readers (p = 0.64).

## Discussion

### Principal findings

Using a representative sample from the Framingham Heart Study cohort, we have demonstrated the feasibility and reproducibility of measuring RPD using CT, a non-invasive marker of papillary calcification, with high intra- and inter-reader correlation.

### In the context of the current literature

CT has been used to assess RPD in prior studies and it has long been known that there is considerable variability in RPD between individuals [13, 14]. It has been demonstrated that RPD is increased in individuals with kidney stones [9, 10, 12] and in those who later develop nephrolithiasis [11], suggesting that it has some utility as a biomarker of kidney stone risk. Although there are no studies directly comparing histologic findings with CT appearance, it has been proposed that the increased density noted in these individuals represents micro-calcification of the papillae which is both known to be associated with an increased risk of kidney stones and is a risk factor for local inflammation, fibrosis and nephron loss [7, 8, 15]. Papillary density has been shown to change in the setting of unilateral ureteral obstruction [16] and malignancy [13] and CT attenuation has been used to distinguish between various histologic subtypes of renal masses [17, 18]. However, techniques to measure RPD are not standardized across studies. Our study seeks to extend the field by demonstrating the reproducibility of a simple technique to measure RPD in a population-based sample.

Using the MDCT study data, other groups from the Framingham Heart Study have described novel, reproducible techniques for measuring renal sinus fat [19], liver fat [20], left atrial size [21] and visceral adipose tissue [22]. The results of our study compare favorably with these results and suggest that this technique could be applied successfully to a larger cohort of individuals in order to investigate a potential role as a biomarker of CKD risk.

### Implications

Our study demonstrates the feasibility of a protocol to measure RPD using CT. RPD may be an important measurement to assess kidney health and may provide important information about an individual’s risk of developing CKD. Future studies will include applying this RPD protocol to a larger sample size to investigate associations between RPD with CVD and CKD risk factors.

### Strengths and limitations

A major strength of this study is our RPD measurement protocol that is easily performed and easily applied to abdominal CT scan data. One limitation is the ethnic homogeneity (primarily non-Hispanic White) of the Framingham Heart Study which may limit the generalizability of our findings to other populations. Another limitation is a lack of renal papillary biopsy to correlate with our RPD measurements. Finally, prior studies have shown that RPD may correlate with hydration status and the urinary specific gravity [16, 23]. No specific protocol was followed for hydration prior to the CT scans and simultaneous urine studies were not performed. Thus we cannot account for the effect of hydration on the results of these measurements.

## Conclusion

We have developed a novel protocol to asses RPD by CT and have shown that this measurement has high intra- and inter-reader reproducibility. Applying this protocol to larger cohorts will provide important information about the utility of this measurement as a biomarker of CKD risk.

## Methods

### Study sample

The participants in this study were derived from the Framingham Heart Study CT sub-study. The Original cohort was first enrolled in 1948. Their children and children’s spouses were enrolled in the Offspring cohort in 1971 and the Third Generation cohort, with at least one parent in the Offspring cohort, were enrolled in 2002 [24, 25]. The Framingham multi-detector computed tomography (MDCT) sub-cohort primarily contains individuals living in the New England area. Participants were qualified if they were female (>40 years old) and not pregnant, male (>35 years old), and had a body weight <160 kg. Between 2002 and 2005, a total of 3539 MDCT participants from the Offspring (n = 1422) and Third Generation cohorts (n = 2117) underwent an MDCT scan as previously described [22]. A sample of 100 participants from the Offspring Cohort were randomly selected for equal representation of age (35–44, 45–54, 55–64, 65–74, and 75–84 years old) and sex strata. Informed consent was required of each participant and this study was approved by the Massachusetts General Hospital and Boston Medical Center Institutional Review Boards and met the terms of the Declaration of Helsinki.

### MDCT scan acquisition

Non-contrast abdominal MDCT scans were captured with an 8-slice MDCT scanner (LightSpeed Ultra, General Electric; Milwaukee, WI, USA) covering 125 mm of the abdomen with 60 consecutive 2.5 mm slices above the S1 level (120kVp, 400 mA, gantry rotation time 500 ms, table feed 3:1). MDCT scans were managed and assessed on the Aquarius 3D Workstation (TeraRecon Inc, San Mateo, CA, USA).

### Protocol development

The abdominal MDCT scans were initially collected to measure abdominal aortic calcification. For this reason, the entire kidneys were not fully visualized in all of our participants. Because the lower position of the right kidney below the liver was better captured by the abdominal MDCT scans, we chose the right kidney for all RPD measurements.

RPD was measured in a single axial slice. In order to choose the optimal slice for reproducibility, we viewed consecutive 2.5 mm slices and selected the image with the largest amount of renal sinus fat. We avoided slices that had obvious kidney stones, neoplasia or cysts. Next, we centered the display on the right kidney and magnified the image to 4×. We then used a measurement tool to draw three ellipses of approximately 20 ± 2 mm^2^. Using the ellipses, we located three areas in the anterior, lateral and posterior part of the slice.

Preference for exact ellipse placement was based on four major criteria. First, preference was given to areas in which there was indentation of the renal papillary tissue into the sinus. Second, we maximized the value of mean Hounsfield unit in the ellipse for each region. Third, we avoided the inclusion of renal sinus fat (−195 to −45 HU range). Finally, ellipses had a minimum distance of at least 5 mm between them, to prevent resampling the same area. We recorded the slice number and the three measurements from the slice. The mean value of the three measurements was reported and used as the final measure going forward.

### Statistical analysis

RPD was measured in a single slice by two readers (GM, AJY) to determine inter-reader reproducibility. The first reader repeated all measurements to assess intra-reader reproducibility. Intra- and inter-reader reproducibility was evaluated with intra-class correlation coefficients. Bland–Altman plots were used to determine any potential systematic bias within the intra-reader and inter-reader repeated measurements. All statistical analyses were conducted using SAS Version 9.2 (SAS Institute Inc; Cary, NC, USA) or STATA Version 12 (StataCorp LP; College Station, TX, USA).

## Abbreviations

CKD: chronic kidney disease
CT: computed tomography
CVD: cardiovascular disease
HU: Hounsfield unit
MDCT: multidetector computed tomography
RPD: renal papillary density
